# The effect of physical activity on Parkinson’s disease outcomes in patients with bilateral subthalamic nucleus deep brain stimulation: a systematic review

**DOI:** 10.3389/fneur.2026.1916520

**Published:** 2026-09-17

**Authors:** Marta Betka, Stanisław Szlufik, Dariusz Koziorowski

**Affiliations:** Department of Neurology, Faculty of Health Sciences, Medical University of Warsaw, Warsaw, Poland

**Keywords:** deep brain stimulation, motor outcomes, Parkinson’s disease, physical activity, systematic review

## Abstract

**Background:**

Subthalamic nucleus deep brain stimulation (DBS-STN) is a recognized treatment for advanced Parkinson’s disease. While it improves some clinical symptoms, especially bradykinesia and rigidity, it has limited application for late motor symptoms. Moreover stimulation-related motor adverse effects can increase the motor burden. There is a clear need to develop additional therapies that complement deep brain stimulation in controlling late motor symptoms. Physical activity is a commonly used and recommended form of therapy for patients with Parkinson’s disease, supplementary to pharmacological therapy. However, there are no clear recommendations including patients after implantation of DBS-STN stimulators in the group undergoing physiotherapy.

**Objective:**

The objective of this study is to systematically review the literature on physiotherapy interventions for patients who have undergone DBS-STN implantation and their influence on motor outcomes.

**Methods:**

This systematic review was conducted in accordance with the Preferred Reporting Items for Systematic Reviews and Meta-Analyses (PRISMA) 2020 guidelines, and a total of 10 studies were included.

**Results:**

Despite heterogeneous physiotherapy protocols, the main outcomes (balance, postural stability, gait speed, functional mobility) improved consistently, while motor severity scores and cognitive or neurophysiological effects remain underexplored.

**Conclusion:**

Current evidence supports the use of physiotheruapy as a potentially effective and safe addition to the management of motor symptoms in patients after DBS-STN. Future trials with standardized physiotherapy protocols and uniform outcome measures are needed to create overall recommendations.

## 1 Introduction

Parkinson’s disease (PD) is a common, progressive neurodegenerative disease. The degeneration of dopaminergic neurons is associated with characteristic clinical symptoms, including resting tremor, rigidity, slowness of movement (bradykinesia), and postural instability. Subthalamic nucleus deep brain stimulation (DBS-STN) is a recognized and widely used treatment for advanced Parkinson’s disease. While it improves clinical symptoms such as tremor, rigidity, and bradykinesia (1–3), it has limited application for late motor symptoms, including akinesia, postural instability, gait, the phenomenon of “freezing,” recurrent falls, balance, and dysarthria (4–7).

Moreover stimulation-related motor adverse effects can increase the motor burden. Daalen et al. investigated the literature for cases of gait or balance worsening after DBS excluding events occurring 1 month after the surgery (to exclude the potential surgery complications) and 12 months after (to exclude the influence of the disease progression). Freezing of gait and falls were the most frequently reported problems, with worsening commonly appearing 3–6 months after surgery. Importantly, the authors argue that postoperative gait/balance deterioration cannot simply be attributed to normal PD progression and may involve stimulation of nearby gait-related pathways (8). Lilleeng et al. researched the short- and long-term impact of DBS implantation on the motor symptoms in PD. They found an initial improvement in UPDRS motor scores and in tremor, bradykinesia, rigidity, postural instability and gait difficulties (PIGD). However, at 6–9 years, all patients had a predominantly PIGD phenotype, and 50% had developed freezing of gait and/or recurrent falls. The authors concluded that long-term progression was particularly characterized by progression of non-dopaminergic motor symptoms (5). An important investigation was performed by Castrioto et al. concerning the long-term outcomes of DBS-STN implantation on PD progression and it separates persistent DBS benefit from progression of Parkinson’s disease. They found that in 10 years, STN-DBS continued to improve overall motor scores, tremor and bradykinesia. However, axial signs showed the greatest progressive loss of responsiveness to both stimulation and levodopa (9). These symptoms reduce the quality of life of patients and make them dependent on their caregivers. There is a clear need to develop additional therapies that complement deep brain stimulation in controlling late motor symptoms.

Physical activity is a commonly used and recommended form of therapy for patients with Parkinson’s disease, used in addition to pharmacological therapy to improve gait, balance, and quality of life (10–12). The clinical practice guideline of American Physical Therapy Association recommends including aerobic exercise, resistance training, balance training, external cueing, community-based exercise, gait training, task-specific training as a highly effective form of physical therapy for patients with Parkinson’s Disease (13). In patients with PD, aerobic capacity is impaired, which leads to reduced endurance of walking and performing activities of daily living (14). Therefore, aerobic exercise, such as cycling on the stationary bicycle or treadmill training, is proven beneficial in improving motor action, gait, and balance (15), as well as reducing disease severity (13). The loss of muscle strength as well as secondary sarcopenia and osteoporosis is another aspect of advanced PD worth considering due to an increase in the risk of falls and reduced quality of life and independence (16–19). Resistance training provided in the graded matter proved beneficial in improving strength, functional outcomes, as well as non-motor symptoms (13). Balance impairment and postural instability are still undertreated symptoms of late PD – balance training is proven as a highly effective form of physiotherapy intervention in improving gait and mobility (13, 20). Gait training remains one of the most important aspects in physiotherapy of patients with PD – gait problems such as reduction in step length, gait speed and freezing of gait phenomenon – remains one of the most challenging disease manifestations (16). There are many forms of gait training that have proven effective in improving these late-motor symptoms, such as treadmill training (21, 22).

Long-term physiotherapy intervention is beneficial for motor symptoms, can delay disease progression and has an impact on dose of parkinsonian medication needed (23, 24). However, there are no clear recommendations for patients after implantation of DBS-STN stimulators concerning physiotherapy (25). To this day, only two randomized clinical trials have been registered assessing the effect of physiotherapy on balance and gait specifically in patients with DBS-STN (26, 27) (Figure 1).

**Figure 1:**
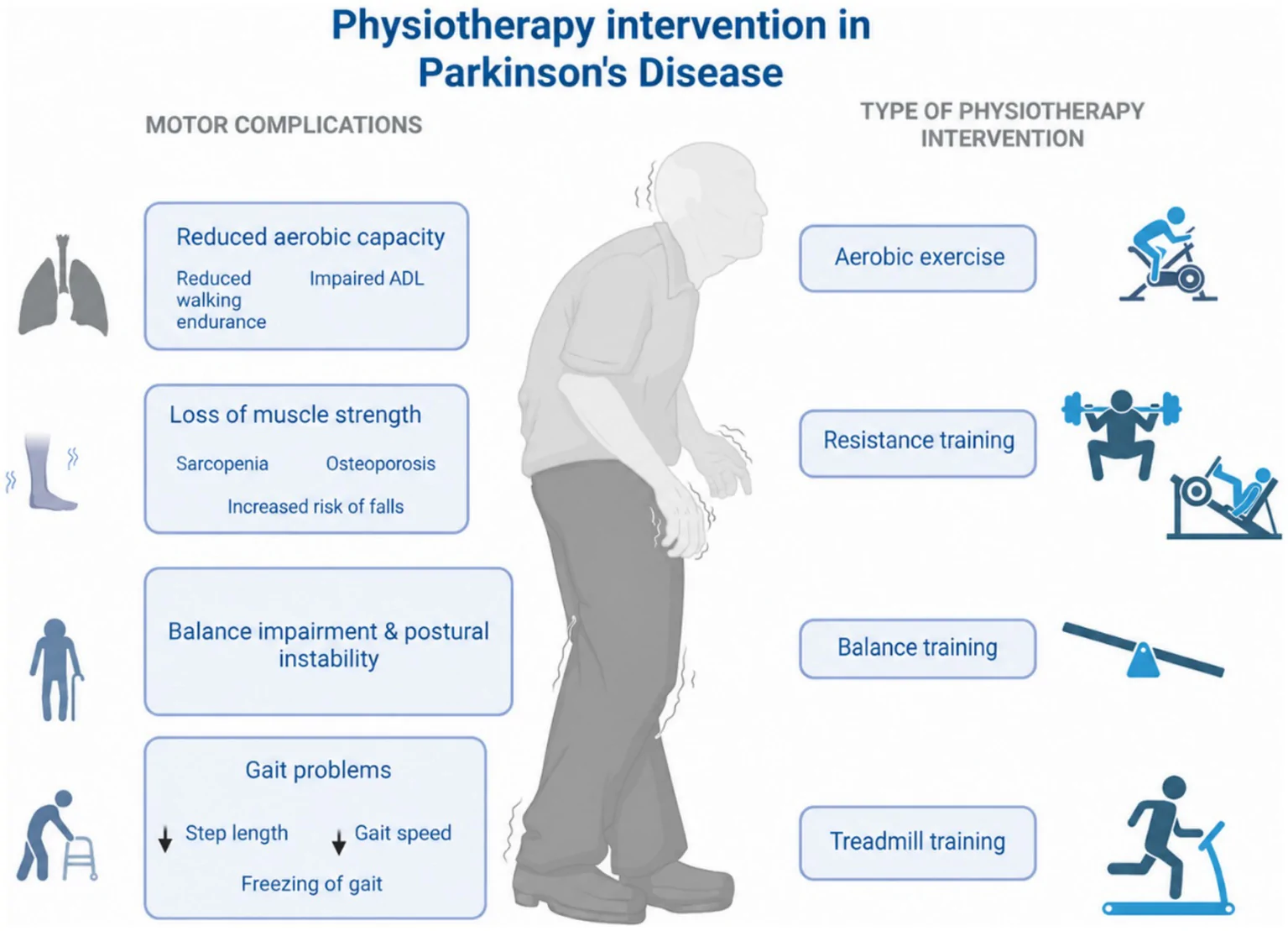
The late motor complications and effective physiotherapy intervention Created with BioRender.com.

## 2 Objective

The aim of this study is to analyze the existing literature on physiotherapy interventions and to systematically assess the motor outcomes in patients who have undergone DBS-STN implantation.

## 3 Methods

### 3.1 Search strategy

This systematic review was done in accordance with the criteria outlined in the Preferred Reporting Items for Systematic Reviews and Meta-Analyses (PRISMA) 2020 guidelines. Institutional Review Board approval and/or patient consent were not required. The computerized search was conducted between January 2000 and January 2025 on the PubMed, Embase, and Web of Science databases. The following algorithm was developed as a search strategy: (parkinson’s disease OR parkinson disease OR parkinsons disease OR pd. OR parkinsons OR parkinsonism) AND (deep brain stimulation OR DBS OR DBS STN OR DBS-STN) AND (physiotherapy OR PT OR physical activity OR physical intervention) AND (motor outcomes OR non-motor outcomes OR physical outcomes OR cognitive outcomes OR quality of life).

### 3.2 Study selection

Studies were included if they met the following criteria: (1) patients with idiopathic Parkinson’s disease, (2) bilateral DBS-STN implantation, (3) physiotherapy intervention, (4) outcomes on motor and/or non-motor symptoms, (5) English language, and (6) peer-reviewed original research with full text. Reviews, systematic reviews, and meta-analyses were excluded.

### 3.3 Data extraction

The following study characteristics were extracted from each included article: (1) author and year of publication, (2) study design, (3) number of patients, (4) sex distribution, (5) disease duration, (6) time since DBS placement, (7) baseline MDS-UPDRS III ON (medication/DBS), (8) baseline Hoehn and Yahr Scale, (9) baseline levodopa equivalent daily dose (LEDD), (10) number of patients with DBS, (11) target of the DBS, (12) comparator group, (13) intervention type, (14) session duration, (15) intervention duration, (16) intervention phases, (17) patient state during intervention, (18) primary outcome parameters, (19) secondary outcome parameters, (20) outcome of the study.

## 4 Results

### 4.1 Search results and study selection

1,120 articles underwent initial screening of titles and abstracts, of which 1,095 were excluded. Subsequently, 25 potentially relevant records were retrieved and assessed for eligibility using a full-text screening process, including the inclusion criteria. Fifteen articles were excluded (*n* = 4 concerning the wrong population, *n* = 9 concerning the wrong type of intervention, *n* = 2 concerning wrong type of outcome). Finally, ten articles met the inclusion criteria and were included in the study. The PRISMA flow diagram of the study selection process is shown in Figure 2.

**Figure 2:**
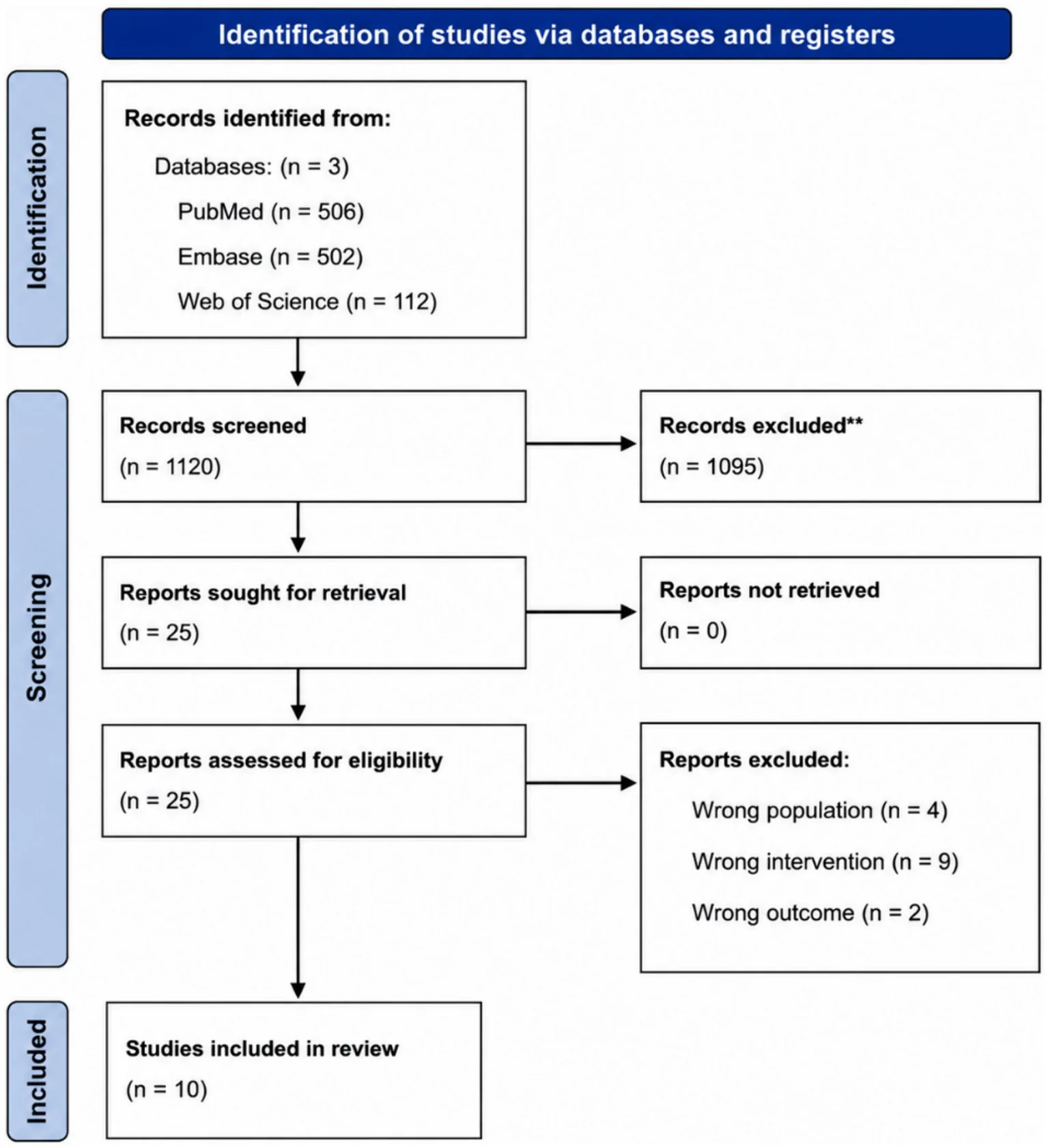
The PRISMA flowchart of the study selection process.

### 4.2 Study characteristics

Ten articles included in the study were conducted between 2009 and 2025. Concerning study designs, two pilot randomized controlled trials (27, 28), two pilot studies (29, 30), two prospective studies (31, 32), two comparison study (33, 34), one retrospective study (35), and one observational study (36) were included. Because the study designs and chosen outcome parameters differed, a quantitative pooled effect size could not be obtained. Instead, we observed the direction of change for each outcome variable across the studies.

### 4.3 Patient demographics

Most of the included studies were small-sample studies with ≤30 participants (ranging from 7 (29) to 60 (33)). All of the studies enrolled middle-aged to older adults (mean ages clustered around the early 60s) with moderate disease duration (7–20 years). Male predominance was observed in most studies. Hoehn and Yahr stages were predominantly II and III, indicating that most participants retained ambulatory ability. Time since DBS placement ranged from a few days (33–35) to several years (27, 29); however, most protocols were initiated > 12 months post-implantation. Differences in time since DBS implantation may have influenced responsiveness to physiotherapy and may substantially affect comparability across included studies. All participants had bilateral STN-DBS. Some studies included comparative groups, but most did not. These demographic data provide context for interpreting the consistent motor benefits (balance, gait) reported across studies, while also emphasizing the need for a larger, more homogeneous population to clarify the impact of age, disease stage, and time of DBS placement on rehabilitation outcomes. The participants’ demographics from the included studies are presented in Table 1.

**Table 1:** Demographics of the participants in the included studies.

Study	Design	Sample size	Age, years	Sex (M/F)	Disease duration, years	DBS duration prior	MDS-UPDRS III	H&Y stage	LEDD, mg	DBS target	Comparator
Tueth et al. (27)	Pilot RCT	29 (EG = 15; CG = 14)	EG: 62.3 ± 5.8 CG: 67.8 ± 7.1	EG: 9/6 CG: 12/2	EG: 12.6 ± 4.6 CG: 11.2 ± 4.9	EG: 47.9 ± 31.2 mo CG: 33.9 ± 27.2 mo	EG: 36.0 ± 13.1 CG: 30.2 ± 8.7	Stage II–IV	NR	Bilateral STN	Yes
Rosenfeldt et al. (29)	Pilot study	7	67 (52–76)	NR	16.1 (10–22)	6.1 (1–14.25) y	40 (25–62)	2.86	427 (300–600)	Bilateral STN	No
Zheng et al. (34)	Non-randomized, controlled, prospective comparative study	24 (EG = 14; CG = 10)	EG: 62.0 ± 9.3 CG: 68.5 ± 4.5	EG: 11/3 CG: 5/5	EG: 7.1 ± 2.9 CG: 9.2 ± 3.8	1 d	EG: 13.9 ± 7.9 CG: 16.5 ± 8.1	NR	EG: 452.7 ± 156.7 CG: 395.0 ± 198.2	Bilateral STN	Yes
Andrade et al. (31)	Prospective controlled	11	61 ± 2	6/5	20 ± 7	20 ± 4 mo	NR	2 ± 1	NR	Bilateral STN	No
Sato et al. (33)	Comparison study	60	60.7 ± 8.9	32/28	12.2 ± 4.6	3 d	18.1 ± 8.6	2.4 ± 0.7	740 ± 272	Bilateral STN	No
Gooßes et al. (28)	Pilot RCT	32	57.6 ± 7.7	24/8	8.6 ± 6.6	NR	NR	NR	NR	NR	Yes
Naro et al. (30)	Pilot study	20 (DBS = 10; non-DBS = 10)	DBS: 62 ± 5 non-DBS: 62 ± 4	DBS: 6/4 non-DBS: 5/5	DBS: 15 ± 2 non-DBS: 14 ± 2	≥12 mo	DBS: 17 (14–21) non-DBS: 28 (27–28)	DBS: 3 (2.5–3) non-DBS: 2.5 (2.5–2.9)	DBS: 740 ± 143 non-DBS: 703 ± 141	Bilateral STN	Yes
Sato et al. (35)	Retrospective	16	61.5 ± 9.5	11/5	13.0 (IQR 8.0)	1–14 d	17.5 ± 7.8	3.0 (IQR 1.0)	1,216 ± 614	Bilateral STN	No
Nardo et al. (32)	Prospective, single-group, pre–post interventional study	9	66.4 ± 6.0	7/2	12.2 ± 6.3	3.1 ± 1.3 y	36.7 ± 6.4	NR	400	NR	No
Tassorelli et al. (36)	Observational	34	57.6 ± 9.4	19/15	11.3 ± 4.4	≤1 mo to >1 y	26.8 ± 12.8	Stages 1–5	NR	Bilateral STN	No

CG, control group; d, days; DBS, deep brain stimulation; EG, experimental group; H&Y, Hoehn and Yahr; LEDD, levodopa equivalent daily dose; mo, months; MDS-UPDRS III, Movement Disorder Society-Unified Parkinson’s Disease Rating Scale part III; NR, not reported; STN, subthalamic nucleus; y, years.

### 4.4 Types of intervention (physiotherapy protocols)

The duration of the rehabilitation programs varied from 8 days (28) to 12 weeks (34); most lasted 4–8 weeks. Session frequency also varied from daily physiotherapy (28, 32, 33, 35) to 2 sessions per week (27, 29, 31). Each physiotherapy session lasted, on average, 60 min. Most protocols included a one-phase intervention without follow-up assessment (one study included a 2-phase intervention (31), and 2 studies included follow-up visits (27, 28)). All programs included balance and gait training. Some of the protocols incorporated additional cognitive load: Naro et al. (30) added rhythmic auditory stimulation (RAS), Gooßes et al. (28) implemented music-driven auditory cueing (MATT), and Rosenfeldt et al. (29) added dual-task exercises. Most of the programs consisted of conventional physiotherapy; however, some included equipment-assisted exercises – for instance, treadmill training (28, 30, 31) or robotic assistance (32). Some of the interventions included home exercises (27). The physiotherapy protocols used in the studies are presented in Table 2.

**Table 2:** Physiotherapy protocols of the included studies.

Study	Intervention	Session duration	Intervention duration	Phases	Patient state
Tueth et al. (27)	Physiotherapy (gait/balance) + home exercise (EG) Control: no PT (CG)	60 min	PT: 16 sessions/8 weeks HE: 16 sessions/8 weeks	1 (+12-week follow-up)	ON medication, ON DBS
Rosenfeldt et al. (29)	DART	45–60 min	16 sessions/8 weeks	1	ON medication, ON DBS
Zheng et al. (34)	Telerehabilitation program	40–60 min	60 sessions/12 weeks	1	ON medication, ON DBS
Andrade et al. (31)	Treadmill training with/without body-weight support	90 min	16 sessions/8 weeks	2	ON medication, ON DBS
Sato et al. (33)	General physical therapy program	40–60 min	14 sessions/2 weeks	1	4 sessions: ON medication/OFF DBS 10 sessions: ON medication/ON DBS
Gooßes et al. (28)	MATT (EG) Ergometer training (CG) Standard rehabilitation (both groups)	20 min	8 sessions/8 consecutive days	1 (+6-week follow-up)	NR
Naro et al. (30)	RAS-assisted treadmill training Conventional physiotherapy	30 min 60 min	24 sessions/4 weeks	1	NR
Sato et al. (35)	Therapeutic exercise	40 min	14 sessions/2 weeks	1	ON medication, ON/OFF DBS
Nardo et al. (32)	RARP	45 min	35 sessions/5 weeks	1	NR
Tassorelli et al. (36)	General exercise program Lee Silverman Voice Therapy	108.3 ± 14.2 min 60 min	20–40 sessions (34.2 ± 15.8 days) 4–5 sessions/4–5 weeks	1	ON medication, ON DBS

DART, dual-task augmented reality treatment; MATT, music-assisted treadmill training; RAS, rhythmic auditory stimulation; CG, control group; DBS, deep brain stimulation; EG, experimental group; HE, home exercise; PT, physiotherapy; RARP, Robotic-Assisted Rehabilitation Protocol; NR, not reported.

### 4.5 Outcome measures and overall trends

Most of the included studies focused on assessing the effects of physiotherapy intervention on balance, postural stability, and gait parameters.

#### 4.5.1 Balance and postural stability

Balance and postural stability were measured with Mini-BESTest, BESTest, and Berg Balance Scale in the majority of the studies – the significant improvement in this domain was shown in E. Tueth et al. (27), Sato et al. (33, 35), Naro et al. (30), Tassorelli et al. (36). Zheng et al. (34) reported improvement in rising from a chair and performing a 360° turn.

#### 4.5.2 Gait

The assessed gait parameters were gait speed, gait distance, and gait velocity; the outcomes were measured primarily using the 2-Minute Walk Test (2MWT) and the 10–Meter Walk Test (10MWT). Improvement, but not significant, was observed by E. Tueth et al. (27) in gait velocity, stride length, and stride length standard deviation when compared to pre- and post-intervention in patients in ON medication, ON stimulation state. Improvement in gait speed under single- and dual-task conditions was observed by Rosenfeldt et al. (29) - more patients showed progress under dual-task conditions. Significant improvement in 10MWT was observed by Naro et al. (30) - greater in patients with DBS (≥ 0.1 m/s). Tassorelli et al. (36) observed improvement of independent walking ability in at least one level in 24/33 patients (73%). Nardo et al. (32) reported significant improvement in gait speed, gait velocity, cadence, stride length and spatiotemporal prameters of the gait after the Robotic-Assisted Rehabilitation program. The 6-Minute Walk Test (6MWD) increased from 351.05 m to 455.57 m in the tele-rehabilitation group in Zheng et al. (34).

#### 4.5.3 Functional mobility

Functional mobility was evaluated with the Timed Up and Go Test (TUG), which is a clinical assessment that measures the time it takes a patient to stand from a chair, walk 3 meters, turn, walk back, and sit down. Improvement in the TUG score was observed by Rosenfeldt et al. (29) under single- and dual-task conditions in all patients, with greater overall improvement in TUG times under dual-task conditions. Andrade et al. (31) reported significant improvements in cognitive TUG scores during treadmill training with and without body weight support, as well as in conventional TUG scores during treadmill training with body weight support. Significant reductions in TUG steps (preoperative vs. at discharge) were observed by Sato et al. (33, 35). Naro et al. (30) declared significant improvement in the TUG score only in patients with DBS after the intervention. The rehabilitation group had improvement in TUG from 17.64 s to 14.28 s in Zheng et al.; follow-up was significantly better than the control group (*p* = 0.002) (34).

#### 4.5.4 Motor severity, activities of daily living and other parameters

Motor severity was rated using the Unified Parkinson’s Disease Rating Scale, Part III (UPDRS-III). Only in four studies was improvement shown; significant improvement was reported by Tassorelli et al. (36) - a reduction in UPDRS-III by 8.3 ± 8.7 points (−27%), *p* < 0.0001, Nardo et al. (32) – a reduction in UPDRS-III by 23.3% in motor scores, 40.7% in the motor UPDRS gait subscore and Zheng et al. (34) – a mean reduction in UPDRS-III by 8.07 points in the rehabiliation group, *p* = 0,000. Sato et al. (33) reported significant improvements in the Trunk Impairment Scale (TIS), StrengthErgo240, and postural sway during closed-eye standing. Significant improvement in Functional Independence Measure (FIM) and Modified Barthel Index (MBI) (*p* < 0.0001) was reported by Tassorelli et al. (36). Zheng et al. (34) reported significant improvement in FVC, FEV1, Borg and PDQ-39 scores in the rehabilitation group. One of the very interesting approaches was presented by Naro et al. (30) - electroencephalographic (EEG) examination was used as an outcome measure and showed stronger β-oscillation remodulation in patients in the DBS group, as well as a β-power increase during the double-support phase. The outcomes of the included studies are presented in Table 3.

**Table 3:** Outcome parameters of the included studies.

Study	Primary outcomes	Secondary outcomes	Balance/postural stability	Gait outcomes	Functional mobility	Motor severity (UPDRS III)	Other outcomes
Tueth et al. (27)	BESTest	Gait velocity; stride length; stride length SD	Significant improvement in BESTest total score.	Improved gait velocity and stride length.	NR	NR	NR
Rosenfeldt et al. (29)	TUG; 2MWT under ST/DT	Gait speed; step length; turn duration	Balance not assessed as primary outcome.	Improved gait speed under single- and dual-task conditions.	Improved TUG performance under ST and DT conditions.	NR	NR
Zheng et al. (34)	UPDRS III, 6MWT, TUG, pulmonary function (FVC and FEV1)	POMA, Tinetti score, Borg scale, PDQ-39	Tinetti score/POMA -Improvement in rising from a chair and performing a 360° turn.	6MWD increased from 351.05 m to 455.57 m in the tele-rehabilitation group.	Significant improvement in TUG.	Significant improvement.	Significant improvement in FVC, FEV1, Borg and PDQ-39 scores.
Andrade et al. (31)	BBS; TUG; static posturography	Cognitive TUG; motor TUG; ML amplitude	No significant change in BBS.	NR	Improved TUG after treadmill training.	NR	NR
Sato et al. (33)	UPDRS III OFF/ON; Mini-BESTest; TUG; TIS	Lower-extremity torque; bradykinesia; postural sway	Significant improvement in Mini-BESTest.	NR	Reduced TUG steps and completion time.	Improved UPDRS III scores.	Improved TIS scores.
Gooßes et al. (28)	UPDRS III; H&Y	GDS; PDQ-39; FIM	NR	NR	NR	NR	NR
Naro et al. (30)	UPDRS III OFF/ON; FES; BBS; TUG; 10MWT	EEG recording	Improved BBS and FES.	Improved 10MWT gait speed.	Improved TUG performance in DBS patients.	Greater improvement in UPDRS III in DBS group.	NR
Sato et al. (35)	UPDRS III OFF/ON; Barthel Index; Mini-BESTest	NR	Improved Mini-BESTest scores.	NR	Improved TUG performance.	No change in UPDRS III.	No change in Barthel Index.
Nardo et al. (32)	Spatio -temporal gait parameters	UPDRS III; kinematic gait parameters; kinetic gait parameters	NR	Gait velocity, cadence, stride length; other spatio -temporal parameters	NR	UPDRS III – significant improvement	3-D gait kinematics and kinetics – no significant improvement
Tassorelli et al. (36)	UPDRS III ON; MBI; FIM; ADL	Standing balance index; walking ability	Improved balance in 17/31 patients.	Improved independent walking ability.	NR	Significant improvement in UPDRS III.	Improved FIM and MBI scores.

2MWT, 2-Minute Walk Test; ADL, activities of daily living; BBS, Berg Balance Scale; DBS, deep brain stimulation; DT, dual task; EEG, electroencephalography; FES, Falls Efficacy Scale; FIM, Functional Independence Measure; GDS, Geriatric Depression Scale; H&Y, Hoehn and Yahr; MBI, Modified Barthel Index; ML, mediolateral; NR, not reported; PDQ-39, Parkinson’s Disease Questionnaire-39; ST, single task; TIS, Trunk Impairment Scale; TUG, Timed Up and Go; UPDRS III, Unified Parkinson’s Disease Rating Scale part III; 6MWT, 6-Minute Walk Test; POMA, Tinetti Performance-Oriented Mobility Assessment.

### 4.6 Safety and feasibility

All studies reported high attendance rates and no serious adverse events, suggesting that even the more intensive daily physiotherapy regimens are well tolerated in the patient population post-DBS (Table 4).

**Table 4:** Safety and feasibility of the studies.

Study	Safety outcomes	Feasibility outcomes
Tueth et al. (27)	No serious adverse events reported.	Mean attendance rate: 93% (≥80% session completion).
Rosenfeldt et al. (29)	No serious adverse events; 2 non-serious events (sciatic pain; non-injurious fall).	Attendance rate: 97.5%.
Zheng et al. (34)	No adverse events reported.	Attendance rate > 60%.
Andrade et al. (31)	No adverse events reported.	Dropout rate: 6/17 participants.
Sato et al. (33)	No serious adverse events reported.	NR
Gooßes et al. (28)	No serious adverse events reported.	Inclusion rate: 84%; completion rate: 60%; dropout rate: 40%.
Naro et al. (30)	No serious adverse events reported.	Mean attendance rate: 100%.
Sato et al. (35)	No adverse events reported.	All participants completed the intervention protocol.
Nardo et al. (32)	NR	NR
Tassorelli et al. (36)	No adverse events reported.	All participants completed the intervention protocol.

NR, not reported.

## 5 Discussion

### 5.1 Heterogeneity of the included studies

The physiotherapy protocols of the included studies varied significantly. One important source of bias in research outcomes in post-operative rehabilitation is the lack of a control group in protocols that assess patients with PD before and after DBS implementation and physiotherapy intervention (33, 35, 36). Therefore, the isolated effect of the postoperative rehabilitation program without the impact of DBS stimulation itself on motor improvement could not be evaluated.

Additionally, the frequency and duration of physiotherapy regimens may influence different motor outcomes: studies that delivered daily, short physiotherapy sessions (28, 33, 35) aimed at rapid, early motor gains, while 8-week, twice-weekly protocols (27, 29, 31) were focused on long-term adaptations. Cueing strategies (visual, auditory, tactile, cognitive) reflect a theoretical approach to improve gait automaticity after DBS; rhythmic auditory stimulation in gait training has proven beneficial for patients with PD (37, 38). Robotic-assisted gait training may improve gait performance and serve as an addition to rehabilitation in patients with Parkinson’s disease who have undergone deep brain stimulation, particularly by increasing gait velocity, cadence, and stride length (32).

Most of the included studies used a combination of balance and gait training, which is needed to control one of the most burdensome late motor symptoms of advanced PD – postural instability. Balance training combined with lower-limb strength training is effective for patients with PD (39). Physiotherapy protocols that included exercises focused on improving range of motion, in addition to gait and balance exercises, improved activity of daily living scores (40). Based on that, it should be considered to include this type of exercise in physiotherapy protocols for patients with PD after DBS-STN in the future.

### 5.2 Consistent motor benefits

Across heterogeneous physiotherapy protocols in the included studies, the main outcomes (balance, postural stability, gait speed, functional mobility) improved consistently, suggesting the efficacy of including patients with PD after DBS-STN in physiotherapy programs. The beneficial effects of physiotherapy intervention on motor symptoms in patients with Parkinson’s disease are well documented. Goodwin et al. (41) reported, in a systematic review with meta-analysis, significant improvements in domains such as balance, gait speed, strength, and health-related quality of life, though the effect sizes varied across included studies. A metanalysis of randomized controlled trials by Tomlinson et al. (42) found pooled motor effects after physiotherapy – significant increase in gait speed (mean difference 0.04 m/s, 95% confidence interval 0.02 to 0.06; *p* < 0.001), TUG test (−0.63 s, 95% confidence interval −1.05 to −0.21; *p* = 0.003), functional reach test (FRT) (2.16 cm, 0.89 to 3.43; *p* < 0.001), and Berg balance scale (BBS) (3.71 points, 2.30 to 5.11; *p* < 0.001) and reduced motor severity in UPDRS-III (−5.01 points, 95% confidence interval −6.30 to −3.72; *p* < 0.001). In the Delphi consensus, Guidetti et al. (43) reported that panelists strongly agreed that PT might improve motor symptoms and quality of life and can be a complementary method to STN stimulation in patients with DBS.

Only two of the included studies involved follow-up visits (27, 28). That reveals the need to include a follow-up assessment after the intervention in studies’ protocols to assess the long-term benefits for patients who underwent DBS-STN implantation. There is also a need to implement home-based physiotherapy programs within a continuum for patients with DBS–STN. The effects of a home physiotherapy regimen for patients with PD were studied by Nieuwboer et al. (44) and were found to be effective in improving scores on the functional activity scale, with a partly sustained improvement during follow-up visits. Zheng et al. (34) reported high adherence rate to the telerehabilitation program and improvement in the functional walking ability and cardiopulmonary fitness in PD patients after DBS.

### 5.3 Underexplored non-motor benefits

In all included studies, the non-motor benefits remained underinvestigated. Costa et al. (45) found that exercise intervention may have beneficial effects for treating non-motor symptoms in Parkinson’s Disease when compared to non-exercise ones. There is a clear need to include investigating the effect of physiotherapy on non-motor symptoms in patients with PD after bilateral DBS-STN.

### 5.4 Neuromodulatory and neuroprotective mechanisms

Another aspect under-researched and worth exploring is the neuromodulatory effect of physiotherapy on patients after DBS-STN implantation. Naro et al. (30) showed stronger β-oscillation remodulation in patients in the DBS group than in the non-DBS group after physiotherapy. Physical activity has proven to be beneficial for patients suffering from neurodegenerative diseases by improving production of neurotransmitters, neurotropic factors, hormones, and promoting neuroplasticity, neurogenesis and angiogenesis (46, 47).

### 5.5 Safety and feasibility

Another important conclusion worth discussing is that despite the intensity of the physiotherapy intervention, no serious adverse effects were observed across the study, indicating that rehabilitation is safe and well-tolerated in patients who had undergone DBS-STN implantation. Moreover, high attendance rates indicate the need and willingness of patients with DBS-STN to participate in such intervention programs.

## 6 Limitations

There are several limitations to this study. First, the number of available studies on the topic is limited. Moreover, the heterogeneity of demographic features (especially the timing of DBS placement prior to intervention) and physiotherapy protocols (program duration, session frequency and length, cueing modality, home exercise implementation, follow-up assessments) makes quantitative analysis difficult. Included evidence base is dominated by small, heterogeneous, mostly non-randomized studies, which limits the strength of any practice recommendations.

## 7 Conclusion

Current evidence supports the use of physiotherapy as a potentially effective and safe adjunct to the management of motor symptoms in patients after DBS-STN. Despite heterogeneous physiotherapy protocols, the main outcomes (balance, postural stability, gait speed, functional mobility) improved consistently, while motor severity scores and cognitive or neurophysiological effects remain underexplored. Future trials with standardized physiotherapy protocols and uniform outcome measures are needed to create overall recommendations.

### 7.1 Clinical key takeaways

- Physiotherapy intervention should be considered as an additional form of therapy in patients after bilateral DBS-STN implantation.
- Physiotherapy protocols may consist of balance, postural stability, gait, and functional mobility exercises.
- Cueing strategies (visual, auditory, tactile, cognitive) could be used as an effective addition to exercise plans.
- Follow-up visits should be considered as part of the physiotherapy protocol.
- A home-exercise plan should be considered as a continuation of physiotherapy intervention.

## Data Availability

Data sharing is not applicable to this article as no datasets were generated or analysed during the current study.

## Glossary

PD: Parkinson’s disease
DBS: Deep brain stimulation
STN: Subthalamic nucleus
PRISMA: Preferred Reporting Items for Systematic Reviews and Meta-Analyses
RCT: Randomized-control trial
EG: Experimental group
CG: Control group
C+: Completers of the rehabilitation program
PT: Physiotherapy
IQR: Interquartile Range
DART: Dual-Task Augmented Reality Treatment
MATT: Music-Assisted Treadmill Training
RAS: Rhythmic auditory stimulation
TUG: Timed Up and Go
2MWT: 2-Minute Walk Test
6MWT: 6-Minute Walk Test
10MWT: 10–Meter Walk Test
ST: Single-task
DT: Dual-task
SUS: Systems Usability Scale
BBS: Berg balance scale
UPDRSIII: Unified Parkinson’s Disease Rating Scale, Part III
MoCA: Montreal Cognitive Assessment
TAP: Test of attentional performance
WMSR: Wechsler Memory Scale, revised version
LPS50+: Leistungsprüfsystem
GDS: Geriatric Depression Scale
PDQ-39: 39-item Parkinson’s Disease Questionnaire
PFS-16: 16-item Parkinson Fatigue Scale
FIM: Functional Independence Measure
FES: Falls efficacy scale
ACE-R: Addenbrooke’s Cognitive Examination–Revised
TIS: Trunk Impairment Scale
MBI: Modified Barthel Index
ADL: Activities of Daily Living
MGHFAC: Massachusetts General Hospital Functional Ambulation Classification
RARP: Robotic-Assisted Rehabilitation Protocol
POMA: Tinetti Performance-Oriented Mobility Assessment
PIDG: Postural Instability and Gait Difficulties
